# High-Efficiency and Reliable Value Geometric Standard: Integrated Periodic Structure Reference Materials

**DOI:** 10.3390/mi14081550

**Published:** 2023-08-01

**Authors:** Chenying Wang, Di Liu, Yaxin Zhang, Weixuan Jing, Song Wang, Feng Han, Qi Mao, Yonglu Wang, Pengcheng Zhang, Zhuangde Jiang

**Affiliations:** 1 School of Instrument Science and Technology, Xi’an Jiaotong University, Xi’an 710049, China; wangchenying@mail.xjtu.edu.cn (C.W.); hanfeng_2062@163.com (F.H.); mq.mq@xjtu.edu.cn (Q.M.); 2 State Key Laboratory for Manufacturing Systems Engineering, International Joint Laboratory for Micro/Nano Manufacturing and Measurement Technologies, Xi’an Jiaotong University, Xi’an 710049, China; liudii@stu.xjtu.edu.cn (D.L.); wangsong2015@stu.xjtu.edu.cn (S.W.); wangyonglu@mail.xjtu.edu.cn (Y.W.); zdjiang@xjtu.edu.cn (Z.J.); 3 School of Mechanical Engineering, Xi’an Jiaotong University, Xi’an 710049, China; 4 The Center for Advancing Materials Performance from the Nanoscale (CAMP-Nano), School of Material Science and Engineering, Xi’an Jiaotong University, Xi’an 710049, China; pczhang2014@xjtu.edu.cn

**Keywords:** integrated, periodic structure, reference materials, line widths, etching depths, sidewall verticality, periods

## Abstract

Integrated periodic structure reference materials are crucial for calibration in optical instruments and micro-computed tomography (micro-CT), yet they face limitations concerning a restricted measurement range, a single pattern type, and a single calibration parameter. In this study, we address these challenges by developing integrated periodic structure reference materials with an expanded measurement range, diverse pattern types, and multiple calibration parameters through a combination of photolithography and inductively coupled plasma (ICP) etching process. These reference materials facilitate high-efficiency and multi-value calibration, finding applications in the calibration of optical instruments and micro-CT systems. The simulations were conducted using MATLAB (R2022b) to examine the structure-morphology changes during the single-step ICP etching process. The variation rules governing line widths, periods, etching depths, and side wall verticality in integrated periodic structure reference materials were thoroughly evaluated. Linewidths were accurately extracted utilizing an advanced image processing algorithm, while average period values were determined through the precise Fast Fourier Transform method. The experimental results demonstrate that the relative errors of line widths do not exceed 17.5%, and the relative errors of periods do not exceed 1.5%. Furthermore, precise control of the etching depth was achieved, ranging from 30 to 60 μm for grids with line widths 2–20 μm. The side wall verticality exhibited remarkable consistency with an angle of 90° ± 0.8°, and its relative error was found to be less than 0.9%.

## 1. Introduction

With the advent of the fourth industrial revolution, microfabrication technologies are increasingly being employed in industrial production, offering significant benefits to manufacturing systems [1,2,3]. Consequently, the demand for micro and nano metrology technology has become apparent [4]. A critical aspect of micro and nano metrology is the calibration of measuring instruments, which relies on traceability and comparison with reference materials [5]. By ensuring the accuracy of the instrument measurements and enabling precise characterization of geometric parameters in micro and nano device structures, calibration plays a pivotal role in enhancing the performance of such devices. Consequently, the development of micro and nano reference materials assumes paramount importance, necessitating the design and fabrication of suitable micro and nanostructures.

**Figure 1:**
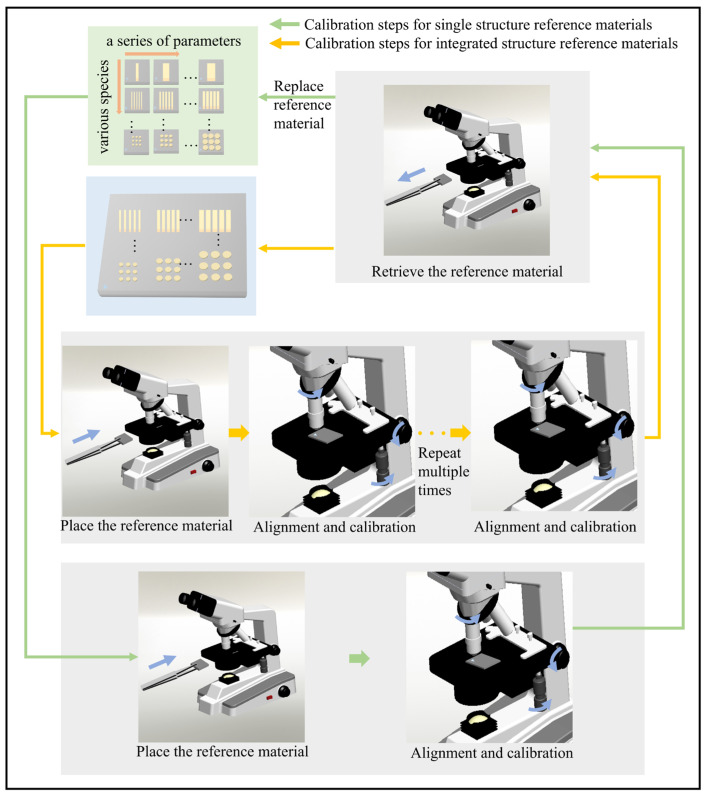
Calibration steps for single structure reference materials and integrated structure reference materials.

In the context of calibrating lenses in optical instruments and micro-computed tomography (micro-CT) systems with varying magnifications, the current practice of employing different reference materials for each calibration scenario leads to low calibration efficiency. Figure 1 illustrates the calibration workflow using single structure reference materials and integrated structure reference materials, shown by the green and yellow arrows, respectively. Measurement errors can arise during the process of replacing reference materials. Integrated structure reference materials offer the advantages of achieving calibration through a single installation, thereby minimizing measurement errors, improving measurement efficiency, and enhancing usability. Integrated structures encompass various forms, such as integrated line widths, integrated grids, and integrated steps. Periodic structures, extensively utilized in electronic circuits and optical devices, assume significant importance within the domain of micro and nanostructures [6,7]. Accordingly, the investigation of micro and nano-integrated periodic structure reference materials holds substantial research significance. The reliable fabrication of such integrated periodic structure reference materials plays a crucial role in ensuring accurate feature parameter transfer, with precise control over micro and nano-integrated periodic structures being a prerequisite.

The National Institute of Standards and Technology (NIST) in the US, Physikalisch-Technische Bundesanstalt (PTB) in Germany, and the National Physical Laboratory (NPL) in the UK have conducted extensive research in this domain [8,9]. Presently, Very Large Scale Integration (VLSI) has successfully fabricated integrated one-dimensional grid reference materials with periods of 1.8 μm, 3 μm, and 5 μm, as well as integrated one-dimensional grid reference materials with periods of 3 μm, 10 μm, and 20 μm, which can be traced back to NIST [10]. However, these integrated one-dimensional grid reference materials are characterized by a limited range of values and are restricted to rectangular grid structures.

Building upon our preliminary research efforts [11,12,13], we have designed an integrated periodic structure reference material incorporating circular and rectangular grids with linewidth feature parameters ranging from 2 μm to 20 μm (2 μm, 4 μm, 6 μm, 8 μm, 10 μm, 15 μm, and 20 μm). These patterns were intelligently integrated within a 3 mm space. In a single preparation process, the incorporation of different feature parameters in the XY direction or the same feature parameters with different graphical structures yields diverse effects. Consequently, achieving controllability of integrated periodic structures with wide scale, multiple pattern types, and multiple parameters proves to present a formidable challenge. Furthermore, critical parameters necessitate evaluation post-preparation. In the accurate measurement of structural parameters, the important parameters include line widths, heights, and sidewall angles [14]. To address these challenges, have implemented a combination of photolithography and inductively coupled plasma (ICP) etching processes to realize controllable fabrication. The etching depths of grids with line widths ranging from 2 μm to 20 μm were meticulously controlled within the range from 30 μm to 60 μm. The relative errors of line widths did not exceed 17.5%, and the relative errors of periods remained below 1.5%. The side wall verticality exhibited an angle of 90° ± 0.8° with a relative error of less than 0.9%. Therefore, successful controllable preparation is achieved. 

## 2. Experiment

**Figure 2:**
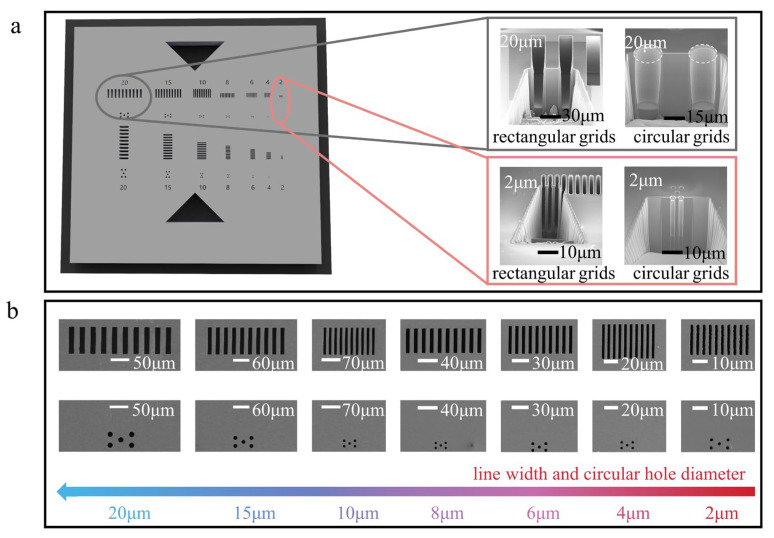
Design of the integrated periodic structure reference materials. (**a**) The three-dimensional structure of the integrated periodic structure reference materials. (**b**) SEM images of periodic structures with 2–20 μm linewidths and circular hole diameter feature parameters.

The experimental investigation focused on the design and fabrication of integrated periodic structure reference materials, comprising tracking structures and calibration structures, as shown in Figure 2a. The tracking structures encompassed triangular and multi-dimensional guides, providing enhanced capabilities for rapid focusing and positioning. The calibration structures consisted of rectangular and circular grids in both horizontal and vertical orientations, facilitating calibration in multiple dimensions without the need for reference material replacement. The spatial resolution of micro-CT is commonly tested using the wire-pair method and the circular hole method. The wire-pair method is suitable for measuring rectangular grids, while the circular hole method is used for measuring circular grids. Therefore, these methods can fulfill the calibration requirements of micro-CT for different measurement techniques. Importantly, the line width feature parameters spanned a wide range, including 2 μm, 4 μm, 6 μm, 8 μm, 10 μm, 15 μm, and 20 μm, as shown in Figure 2b. This parameter design catered to the measurement range requirements of different measuring devices, such as optical microscopes and micro-CT systems. Each rectangular grid, for every feature parameter, comprised ten rectangular individual structures with a 50% duty cycle. Similarly, each circular grid, for every feature parameter, consisted of five circular individual structures. Notably, the circular grids positioned at the lower side of the horizontal rectangular grids exhibited a 25% horizontal duty cycle and a 50% vertical duty cycle.

The fabrication process involved a meticulously designed combination of photolithography and ICP etching, enabling precise pattern transfers. Initially, silicon oxide wafers, composed of N-type Si (100) with a thickness of 500 μm and a 300 nm SiO_2_ layer, were prepared through thermal oxidation on silicon wafers. Subsequently, a series of pre-treatment steps encompassing cleaning and drying were performed on the silicon oxide wafers. The photoresist (EPG535, Taiwan, China) was applied to the silicon oxide wafers using a spin coater (KW-4A, SETCAS Electronics Co., Ltd., Beijing, China). The spin coating process involved initially setting the low speed to 500 rpm for 5 s, followed by setting the high speed to 3000 rpm for 20 s. Once the spin coater was set up, the photoresist was uniformly and gently dripped onto the surface of the silicon oxide wafers using a dropper, and the spin coating procedure was completed based on centrifugal force. The thickness of the photoresist layer was accurately measured as 1.2 μm using an optical interferometry film thickness gauge (TohoSpec 3100, Toho Technology, Aichi, Japan). A photoresist pattern incorporating the integrated periodic structures was then generated using a sophisticated photolithography system (ABM6, ABM, Inc., New York, NY, USA). The exposure time is 7.3 s, and the exposure dose is 219 mJ/cm^2^. Subsequently, the silicon dioxide layer was selectively etched using the ICP etching process (DSE200S, NAURA Technology Group Co., Ltd., Beijing, China) to form a double-layer mask comprising photoresist and silicon dioxide. The subsequent etching of the silicon substrate was carried out utilizing the ICP etching process with precisely controlled parameters, including 80 etching cycles. SF_6_ gas was employed as the etching gas, with a flow rate of 250 sccm, while C_4_F_8_ gas served as the passivation gas at a flow rate of 180 sccm. The passivation etching time ratio was 1.2/3.5, and the pressure during the etching process was carefully regulated at 60 mTorr. The RF power employed during etching and passivation was set at 2200 W and 1800 W, respectively. Finally, the photoresist layer was effectively removed using a combination of acetone and anhydrous ethanol. To ensure the thorough elimination of photoresist residue, a meticulous cleaning step involving a 3:1 mixture of sulfuric acid and hydrogen peroxide was implemented on the fabricated reference materials.

The three-dimensional morphologies of the fabricated integrated periodic structure reference materials were comprehensively characterized using a laser confocal microscope (OLS4000, Semicon, Tokyo, Japan). This advanced imaging technique facilitated the accurate assessment of crucial parameters, including periods, etching depths, and sidewall verticality. Furthermore, the surface structures of the fabricated reference materials were examined using a scanning electron microscope (SU-8010, Hitachi, Tokyo, Japan) to evaluate linewidths.

## 3. Results and Discussion

To ensure the reliable quality assessment of the fabricated integrated periodic structures reference materials, comprehensive and accurate characterization of key parameters, including line width, period, depth, and sidewall verticality, was performed. The integrated periodic structure reference materials underwent rigorous analysis through advanced image processing algorithms [15], resulting in the extraction of horizontal rectangular grid edges and subsequent line widths calculations. The image processing workflow involved several steps to achieve accurate linewidth extraction. Firstly, binarization was performed using the maximum inter-class variance method. This method calculates the threshold value that maximizes the difference between the class variances of the foreground and background pixels, resulting in an optimal separation of the image into binary regions. Next, closed operations were applied to remove small gaps or holes within the binary regions. This step helps to smooth the binary contours and ensure the integrity of the desired features. After that, median filtering was employed to reduce noise and further enhance the quality of the binary image. The median filter replaces each pixel with the median value of its neighboring pixels, effectively reducing the impact of outliers or small-scale variations. Finally, edge extraction techniques were utilized to detect and extract the edges of the features of interest. These techniques identify abrupt changes in pixel intensity and generate a binary edge map, which provides precise information about the boundaries of the desired features. 

**Figure 3:**
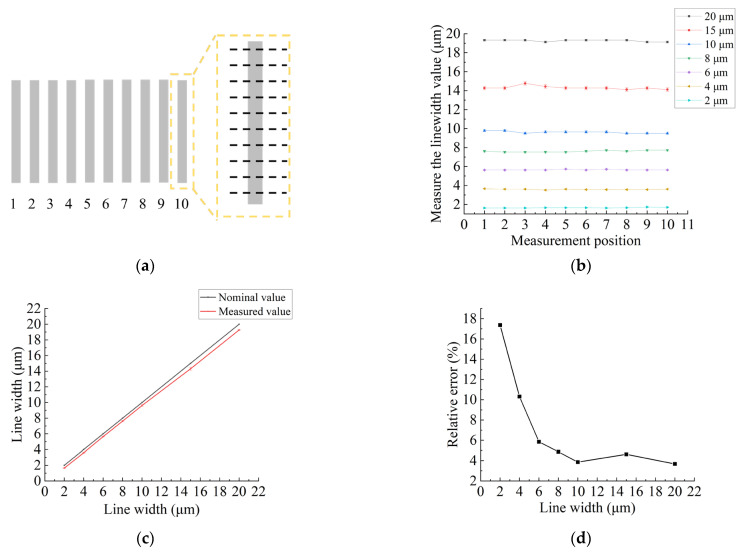
Measurement results of 2–20 μm line widths. (**a**) Measurement positions of line widths. (**b**) Measurement results for 10 measurement positions of 2–20 μm line widths. (**c**) Comparison between the measured values and the nominal values of 2–20 μm line widths. (**d**) The relative errors of measured values with 2–20 μm line widths.

Figure 3a shows the uniform selection of ten positions along the grid line, with the average line widths of these positions serving as the measured values for the corresponding grid lines. In Figure 3b, each position corresponds to each grid line, and each coordinate point represents the average of ten measured values on that grid line. The line width measurement outcomes for grids ranging from 2–20 μm are shown in Figure 3b. A notable observation from the line width measurements is the increasing disparity between the measured values and the nominal values as the line widths increase, as shown in Figure 3c. However, a gradual decrease in relative errors is observed in Figure 3d. This finding suggests a diminishing controllability of line width as it decreases. The first reason is that as the line width decreases, fabrication becomes increasingly challenging due to limitations in manufacturing techniques or equipment, such as the resolution constraint of lithography systems. The second reason is that introducing errors in determining the threshold through image processing algorithms can lead to the reduction of line width values. 

Two approaches can be considered to address these limitations and minimize relative errors. The first approach is to improve the fabrication process itself by utilizing manufacturing techniques that are better suited for small line widths. The second approach involves algorithmic compensation during graphic processing. This means that instead of relying solely on the image processing algorithms to determine the threshold value, additional compensation or correction algorithms can be applied to mitigate potential errors. These compensation algorithms can take into account factors such as noise, variations in contrast, or other image artifacts that may affect the accuracy of the threshold determination. By incorporating such compensation techniques, the relative errors introduced during the image processing stage can be reduced, leading to more accurate and reliable results.

**Figure 4:**
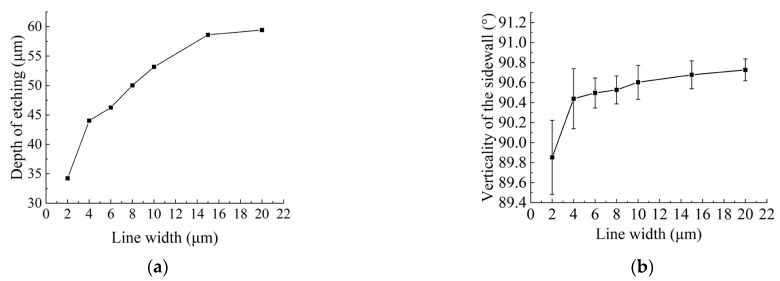
Evaluation of etching depth and sidewall verticality. (**a**) The relationship between line widths and depth of etching. (**b**) The relationship between line widths and verticality of the sidewalls.

To ensure the accurate and reliable value of a quantity transfer, the investigation also involved the calculation of etching depths and sidewalls verticality for integrated periodic structure reference materials with line widths ranging from 2–20 μm. Figure 4a demonstrates a corresponding increase in etching depths with increasing line widths, effectively controlled within the range of 30–60 μm. Notably, a correlation between the etching rates and depths is observed due to the fixed number of cycles in the ICP etching process. This indicates that during the preparation of integrated reference materials, the etching rate varies with the variation of line width. As the line width decreases, the etching rate slows down. The size of the etching channel is determined by the line width, and a smaller line width corresponds to a narrower etching channel. Consequently, fewer etching particles enter the channel, resulting in a reduced etching rate and a corresponding decrease in the etching depth. Additionally, the narrow etching channel hinders the transportation of etching products and reactants, further contributing to a decline in etching capacity and a reduction in the etching rate. Therefore, under the same etching time, the etching depth decreases as the line width decreases.

Figure 4b reveals a decrease in sidewall angles as line widths decrease, constrained to 90° ± 0.8°, with relative errors below 0.9%. This phenomenon indicates that when etching particles enter the narrow channel, an increasing lateral etching effect on the sidewalls, which influences their verticality. The diminished line widths result in a more pronounced impact on etching depths and sidewall verticality. This behavior can be attributed to the higher probability of scattering and re-scattering of etched ions within narrow channels. Among them, the etching depth of 2 μm line width exceeds 30 μm, and the depth-to-width ratio exceeds 1:15, which is the largest depth-to-width ratio and the smallest etching channel in 2–20 μm line width in this study. During the etching process, as the etching depth increases, the processes of etching particle scattering and re-scattering increase. Consequently, they continually react with the silicon substrate on the sidewalls, leading to a reduction in sidewall verticality.

**Figure 5:**
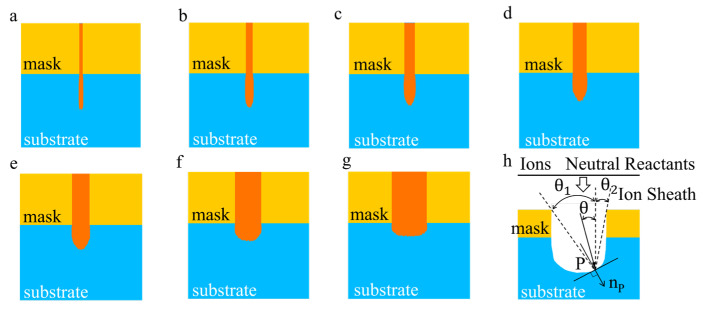
Simulation results of single-step etching morphology of 2–20 μm line widths, where (**a**) 2 μm, (**b**) 4 μm, (**c**) 6 μm, (**d**) 8 μm, (**e**) 10 μm, (**f**) 15 μm, and (**g**) 20 μm line width. The schematic diagram of the etched geometric model is shown in (**h**).

To gain insights into the theoretical aspects of ICP etching morphology, a MATLAB-based simulation model was developed. During the ICP etching process, the passivation and etching procedures were executed alternately, which was the result of a combination of physical bombardment and chemical reactions as well [16,17]. The ICP etching process was simplified to a single-step etching scenario to facilitate a clear observation of etching morphology. Due to the high etching selection ratio of substrate silicon and mask silica, ICP etching of the mask is ignored in the simulation. In Figure 5h, *θ*_1_ − *θ*_2_ is the etching window of point P. The etching process in plasma is influenced by two main factors: ions and neutral particles. Among these, the ratio of neutral particles to ions is 1:10. By converting the quantity ratio into probabilities, each particle has a 1/11 chance of being emitted as an ion and a 10/11 chance of being emitted as a neutral particle. The incident ions undergo acceleration in the sheath field due to the applied electric field. Considering the sufficiently low gas pressure, it is assumed that there are no collisions, and the incident ions acquire energy within this field. Additionally, due to the ionized gas having a certain temperature, the ions possess initial thermal motion energy and angles. The incident angle is determined by the ratio of the sheath voltage to the ion temperature. And it was found to deviate from perfect perpendicularity to the substrate surface, following a probability distribution function modeled by a normal distribution [18]. The distribution allowed for the generation of random emission angle values. The etching process was refined to accurately reflect real-world conditions by considering the probabilities of various reactions [19]. 

Building upon the model, we consider a substrate consisting of a 70 × 100 array of silicon atoms. The corresponding mask window is designed to accommodate line widths ranging from 2 to 20 μm. Furthermore, to observe the morphological changes, an equal number of etched ions is employed for each line width. The simulated results presented in Figure 5a–g demonstrate a progressive reduction in the flatness of the etched bottom and an intensified lateral etching on the sidewalls as the line widths decrease. These findings align with the etching morphology depicted in Figure 2a. In the simulation, an equal number of etched particles with line widths ranging from 2–20 μm were used. As the etching process progressed and all particles were consumed, it was observed that the etched depth increased as the line width decreased. This indicates that in practical etching, the number of particles entering the etched channels with different line widths is not uniform, resulting in a reduction in etching depth as the linewidth decreases.

Furthermore, the periods of the integrated periodic structure reference materials were measured using a laser confocal microscope and calculated using the Fast Fourier Transform method [3]. The Fast Fourier Transform (FFT) technique is utilized to process the scanned cross-section profile data of the grid structure, resulting in its spectrum diagram. The grid period value can be determined as 1/f_max_, where f_max_ represents the frequency corresponding to the highest peak value in the spectrum diagram. Thus, measuring the grid period value is equivalent to determining the value of f_max_. The FFT method offers several advantages for calculating the grid period, including its ability to handle diverse surface topographies and its efficiency in quickly computing the periodic grid period value. Ten positions were uniformly selected along the length direction of the rectangular grids, and their average values were calculated as evaluation values for the corresponding periods. Relative errors were also calculated using Equation (1). The relative errors (δ) can be expressed as

(1) δ=Δ/L×100%

where $\Delta =|L_{0}-L|$ represents the absolute error, *L* denotes the nominal value, *L*_0_ represents the measured value.

The obtained evaluation values for line widths, periods, relative errors of line widths and periods, depths of etching, and sidewall verticality were compiled and summarized in Table 1. The reference materials exhibited period relative errors of below 1.5%. The relative error for line widths of 6–20 μm is less than 6%, while the relative error for line widths of 2 μm and 4 μm is less than 17.5%. The structural depths ranged from 30 μm to 60 μm, while the grid sidewalls demonstrated a verticality of 90° ± 0.8°. These results provide strong evidence of the successful controllability achieved during the preparation of integrated periodic structure reference materials.

## 4. Conclusions

In this study, we address the limitations of narrow measurement range and single pattern type in integrated periodic structure reference materials. We successfully fabricate integrated periodic structure reference materials with linewidth characteristic parameters ranging from 2 μm to 20 μm. The obtained values for these parameters are reliable, and the relative errors of line widths, periods, and sidewall verticality are effectively controlled within a small range. Specifically, the linewidth relative errors are limited to 17.5%, while the period relative errors do not exceed 1.5%. Furthermore, the verticality of the grid sidewall is 90° ± 0.9°. The relative errors of grid sidewall verticality remain below 0.9%. Additionally, the grid depth ranges from 30–60 μm. As the line widths decrease, the relative errors of the line widths gradually increase, and the depths of etching and the angles of the sidewalls decrease accordingly. In narrow channels, the transportation of etching reactants and products becomes more challenging and has a higher probability of etching particles scattering and re-scattering. This phenomenon leads to more pronounced lateral etching effects. Consequently, when the line width is smaller, the etching depth and sidewall verticality are more significantly affected. MATLAB simulations have confirmed that as the line width decreases, the lateral etching of the sidewall intensifies, leading to a decrease in the verticality of the grid sidewall.

Optical microscopy employs reference materials that provide comprehensive and accurate characterization for calibration, ensuring precise measurements of micro and nanodevices and facilitating the manufacturing process. This calibration process greatly benefits the accurate measurement and fabrication of micro and nanodevices. An additional advantage of these integrated periodic structure reference materials is their capability for multiple measurements without the need for reference material replacement. This characteristic not only reduces measurement errors but also enhances measurement efficiency. These reference materials offer a valuable tool for calibrating various optical instruments and micro-CT systems. As a result, they find broad applications in the field of calibration. High efficiency and reliable value have always been the development direction of integrated reference materials. 

## Figures and Tables

**Table 1:** The evaluation values of line widths and periods, the relative errors of line widths and periods, depths, and sidewall’s verticality of the integrated periodic reference materials. (Unit: μm).

Nominal line width	2	4	6	8	10	15	20
Measured line width	1.65	3.59	5.65	7.61	9.61	14.31	19.26
Relative error of line width	17.50%	10.25%	5.83%	4.88%	3.90%	4.60%	3.70%
Nominal period	4	8	12	16	20	30	40
Measured period	3.94	7.89	11.84	16.02	20.30	30.45	39.96
Relative error of period	1.50%	1.38%	1.33%	0.13%	1.50%	1.50%	0.10%
Measured depth	34.23	44.02	56.25	50.02	53.16	58.61	59.43
Verticality of sidewall	89.85°	90.44°	90.50°	90.53°	90.60°	90.68°	90.73°

## Data Availability

Data sharing does not apply to this article.
